# Case report: Long-term clinical benefit of pyrotinib therapy following trastuzumab resistance in *HER2*-amplification recurrent mucinous ovarian carcinoma

**DOI:** 10.3389/fonc.2022.1024677

**Published:** 2022-12-22

**Authors:** Xiangming Fang, Haibo Mou, Xinxin Ying, Xuehua Hou, Luo Wang, Ying Wu, Naimeng Yan, Lijie Guo, Qin Liao

**Affiliations:** ^1^ Department of Medical Oncology, Shulan (Hangzhou) Hospital Affiliated to Zhejiang Shuren University, Shulan International Medical College, Hangzhou, China; ^2^ Medical Department, OrigiMed Co., Ltd, Shanghai, China

**Keywords:** *HER2* amplification, trastuzumab, pyrotinib, mucinous ovarian carcinoma, next-generation sequencing

## Abstract

Advanced or recurrent mucinous carcinoma of the ovary minimally responds to current cytotoxic treatments and has a poor prognosis. Despite multimodal treatment with chemotherapy and surgery, most patients ultimately progress and require palliative systemic therapy. Anti-HER2 therapy has been demonstrated to be an effective strategy for the treatment of HER2-positive breast cancer. However, the role of anti-HER2 therapy in ovarian cancer remains largely unknown. Here, we report the case of a young woman with FIGO Stage IIIc recurrent mucinous ovarian carcinoma (MOC) who developed trastuzumab resistance and disease progression following cross-treatment with trastuzumab combined with pertuzumab. *HER2* amplification was discovered using next-generation sequencing (NGS). The patient then received bevacizumab, and pyrotinib (an irreversible HER2 antagonist) plus capecitabine treatment, and achieved a long-term clinical benefit for 22 months. Pyrotinib combined with bevacizumab is a potential treatment for MOC patients who are heavily pretreated and harbor a *HER2* amplification. Our case may provide valuable treatment information for patients with advanced or recurrent MOC.

## Introduction

Mucinous ovarian carcinoma (MOC) is a rare, histological subtype tumor, accounting for approximately 3% of all epithelial ovarian cancers (1–3). At 5 years following diagnosis, the corresponding proportions for FIGO stage I, II and III/IV MOC patients were 91.1%, 76.7% and 19.8% (4). Furthermore, 5-year survival for patients with stage III MOC was 25.7%, and for patients with stage IV MOC was 10.2% (5). Advanced MOC has a poorer prognosis than other histological subgroups, and often poses a therapeutic challenge for oncologists. At present, the complete surgical resection of MOC is considered gold-standard treatment. Patients with advanced MOC tend to be more resistant to standard chemotherapy and have a worse prognosis compared to other epithelial subtypes (6). Adjuvant platinum-based chemotherapy is generally used for advanced disease and is more suitable for high-grade serous ovarian cancer, although it has low therapeutic efficacy in regard to MOC (7). Current targeted therapies for the treatment of advanced ovarian cancer include anti-angiogenic agents and PARP inhibitors (8, 9). Due to the heterogeneity of MOC and poor patient prognosis, more targeted therapies need to be explored and their application in ovarian cancer needs to be clinical evaluated (10, 11). Increased HER2 (ERBB2) expression is associated with poor survival and a resistance to platinum therapy, and its inhibitors have also been supported by preclinical data. Therefore, the potential for HER2-targeted therapy is on-going (12, 13). In the past, targeting HER2 has been a key therapeutic strategy in HER2-positive breast cancer, and such a strategy may also have promise for the treatment of HER2-positive ovarian cancer. In this study, we report a case of recurrent MOC harboring *HER2* amplification progression following a trastuzumab-based regimen. Our results indicated a significant benefit from pyrotinib and bevacizumab, as well as a long-term clinical benefit over 23 months due to continued pyrotinib and bevacizumab treatment. As mentioned above, patients with advanced recurrent MOC display significant risk for recurrence/death. Therefore, the treatment regimen in this case is expected to yield positive results in large-scale clinical studies aimed at developing additional treatments for MOC.

## Case report

Our patient was a 23-year-old female with a history of bilateral ovarian cystectomy due to lower abdominal pain and torsion of ovarian cyst pedicle, an omentectomy and appendectomy between November 2018 and August 2019. Due to nausea and abdominal distension, the patient visited the Zhoushan District Maternity and Child Health Care Hospital on February 4, 2020. Enhanced computed tomography (CT) of the entire abdomen revealed a solid mass within the right adnexal area (10.3 × 8.2 cm). On February 21, 2020, the patient was referred to our hospital for further examination. Positron Emission Tomography-CT (PET-CT) indicated a large pelvic mass and increased FDG metabolism. Based on the patient’s medical history, an ovarian malignant tumor was thought to have re-occurred and to be locally adhered to the uterine roof, and pelvic peritoneal seeding metastasis was also considered.

**Figure 1:**
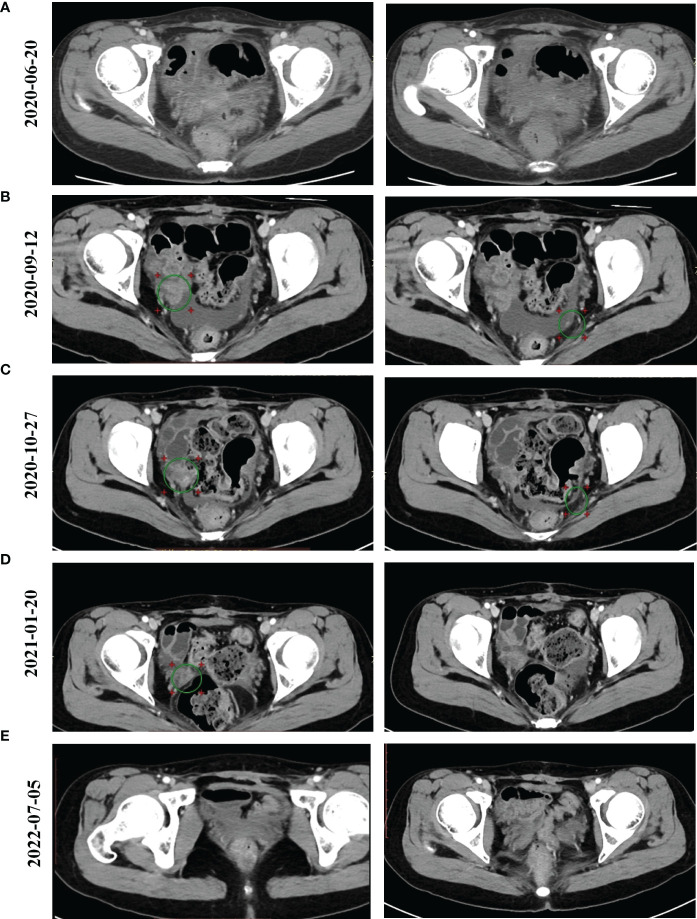
Computed Tomography (CT) images for our patient over the course of treatment. **(A)** The results of abdominal enhanced CT scan for the patient following two cycles of trastuzumab/pertuzumab/capecitabine therapy after the patient underwent radical surgery on 20 June 2020. **(B)** The results of abdominal CT scan for the patient following six cycles of trastuzumab/pertuzumab/capecitabine therapy on 12 September 2020. **(C)** The results of abdominal CT scan for the patient following two cycles of pyrotinib/bevacizumab/capecitabine therapy on 27 October 2020. **(D)** The results of a PET-CT scan for the patient following six cycles of pyrotinib/bevacizumab/capecitabine therapy on 20 January 2021. **(E)** Positron Emission Tomography-CT (PET-CT) scan showed our patient’s condition on 5 July 2022.

**Figure 2:**
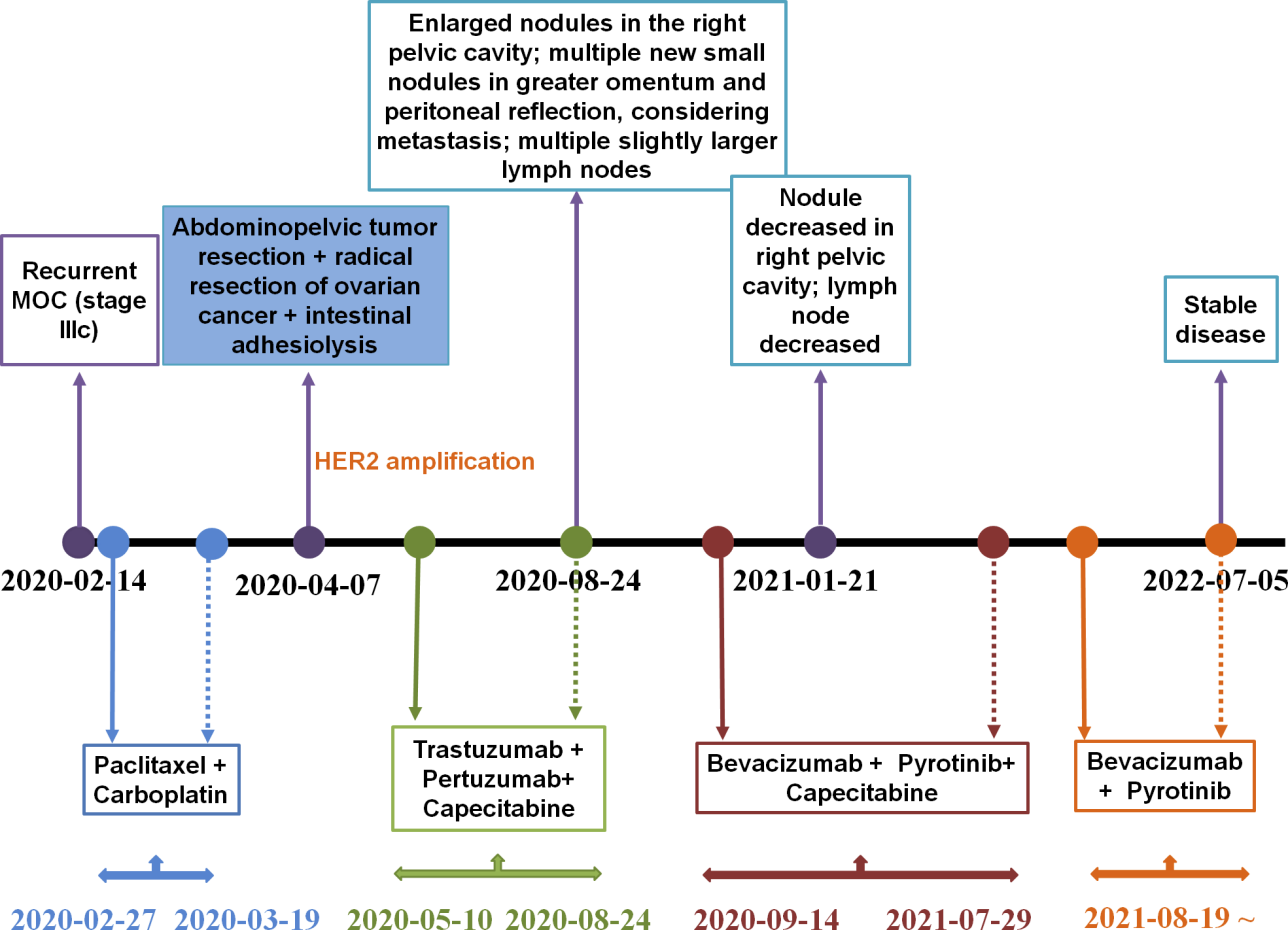
A summary of the patient’s treatment regimen.

From February 27, 2020 until March 19, 2020, the patient received two cycles of a TcP regimen (paclitaxel at 240 mg and carboplatin at 700 mg on day one, every three weeks) as neoadjuvant chemotherapy. The patient then underwent radical resection for ovarian cancer and an abdominal pelvic tumor resection on April 7, 2020. Immunohistochemistry (IHC) results indicated CA125 (+), caudal-related homeobox transcription factor 2 (CDX2) (+), cytokeratin 19 (CK19) (+), CK20 (+), CK7 (+), CK8/18 (+), E-cadherin (+), p53 (30%+), Ki-67 (30%+), estrogen receptor (ER) (−), NapsinA (–), progesterone-receptor (–), Wilms tumor-1 (WT-1) (–), and carcinoembryonic antigen (–). Based on results obtained from the histopathological and IHC analyses, the tumor was diagnosed as a highly focal invasive differentiated MOC with recurrent Stage IIIc according to the International Federation of Gynecology and Obstetrics (FIGO). Tumor tissues were sent for next generation sequencing (NGS) testing, and *HER2* amplification, *TP53* R175H, *KRAS* G12A, *AXIN1/COQ9* rearrangement and *AXIN1/CCDC102A* rearrangement were identified using a 706 gene panel. One month following surgery, an abdominal enhanced CT scan revealed extensive thickening of the lower abdominal peritoneum and mesentery, irregular encapsulated effusion in the pelvic peritoneal reflection, and multiple slightly larger lymph nodes in the hepatogastric ligament, the portacaval space, the mesangial area, and the pelvis. Adjuvant therapy with trastuzumab (168 mg), plus pertuzumab (840 mg) and capecitabine (morning: 1g, evening: 1.5g), was then administered every three weeks. After three cycles of adjuvant therapy, the abdominal enhanced CT scan revealed decreased mesoperitoneal thickening, decreased abdominopelvic effusion; and smaller lymph nodes in the hepatogastric ligament, the mesangial area, the ileocecal junction, and the pelvic cavity as compared to pretreatment (**Figure 1A**). Following six cycles of adjuvant therapy with trastuzumab, plus pertuzumab and capecitabine, CT images of abdomen showed nodules on the right side of the pelvic cavity larger than previously identified, and observed multiple new nodules in omental and peritoneal reflection, which were considered to be metastases; and the hepatogastric ligament, the mesangial area, the ileocecal junction, and the pelvic nucleus contained multiple, slightly larger inguinal lymph nodes (on both sides, **Figure 1B**). By considering that disease progression may be the reason for acquired resistance to dual anti-HER2 antibody therapy with trastuzumab and pertuzumab, pyrotinib was prescribed on September 14, 2020. The patient was then treated with bevacizumab (300 mg), pyrotinib (160 mg) and capecitabine (1g, bid), every three weeks from September 14, 2020. Following three cycles of treatment, abdominal CT scan revealed decreased pelvic effusion, and small nodules in the original peritoneal reflection that were not obvious, as compared to the time of regimen change on January 21, 2021 (**Figure 1C**). After three additional cycles of treatment, the Positron Emission Tomography-CT (PET-CT) showed the right pelvic nodule once again regressed (**Figure 1D**). On July 5, 2022, abdominal CT scan revealed scattered small lymph nodes in the hepatogastric ligament, the mesangial area, the retroperitoneum, and the bilateral inguinal region, suggesting the disease has no progression (**Figure 1E**). Until the time of publication, pyrotinib and bevacizumab have been administered as a maintenance therapy, the patient’s lesions are still stable, and no recurrence or metastasis has occurred within 22 months. **Figure 2** provides a timeline that includes relevant patient care data and treatment regimen.

## Discussion

Several past studies have indicated that patients with advanced MOC have a less favorable prognosis than patients with advanced serous ovarian cancer (14). MOC is an uncommon histological type of ovarian cancer that poorly responds to conventional chemotherapy regimens. In this study, we reported the case of a patient harboring *HER2* amplification recurrent MOC, diagnosed as FIGO Stage IIIc, who achieved a significantly longer progression free survival (PFS) than expected using anti-HER2 targeted therapy with pyrotinib and bevacizumab, following resistance to dual anti-HER2 antibodies with trastuzumab and pertuzumab. Our results suggest the promising efficacy of pyrotinib as an anti-HER2 targeted therapy in the treatment of recurrent MOC with *HER2* amplification.

HER2 is a member of the epidermal growth factor family of tyrosine kinase receptors. Functional activation of HER2 promotes oncogenesis, which has led to the investigation of HER2-directed agents in cancers with *HER2* alterations (15). HER2 overexpression has already been reported in several types of cancer, although the benefits of targeted therapies are variable and anecdotal (16), and likely depend on the levels of HER2 expression, the presence of tumor heterogeneity, and the activation status of other pathways. Current HER2-directed therapies include monoclonal antibodies, antibody-drug conjugates, and small molecule tyrosine kinase inhibitors. The monoclonal antibodies trastuzumab and pertuzumab, respectively, bind to distinct regions of *HER2* extracellular domain (ECD) at juxtamembrane domain IV and dimerization domain II, thereby inhibiting HER2 activity through cell-intrinsic effects and promoting antibody-dependent, cell-mediated cytotoxicity (17–19). Previous studies showed *HER2* amplification in ~20% of MOC cases (20), and *HER2* is known to be involved in the pathogenesis of MOC (21).Trastuzumab therapy is a treatment option for patients with mucinous carcinoma when a tumor has *HER2* amplification and overexpression (20), although the clinical response to the single agent trastuzumab in epithelial ovarian cancer has been disappointing (22). Faratian et al. (23, 24) suggested that trastuzumab, in combination with pertuzumab, may be an effective approach for high HER2-expressing ovarian cancers and may also enhance sensitivity to endocrine therapy in ERα-positive ovarian cancer. A Caucasian woman diagnosed with advanced high-grade serous ovarian carcinoma with focal amplification for the *HER2* gene displayed a durable partial response to pertuzumab and trastuzumab without concomitant chemotherapy (24). Significant responses were also observed in one patient with *HER2*-amplified recurrent MOC who received trastuzumab in combination with conventional chemotherapy, following the discontinuation of conventional therapy (20). For our case, the patient with *HER2*-amplified recurrent MOC was treated with trastuzumab plus pertuzumab in combination with chemotherapy. However, disease progressed followed six cycles of treatment. Previous studies suggested that the *HER2* mutation may be a critical reason for resistance and disease progression in patients treated with anti-HER2 monoclonal trastuzumab or dual anti-HER2 antibodies using trastuzumab and pertuzumab (15, 25). Therefore, we speculate that the *HER2* mutation is also associated with resistance to trastuzumab and pertuzumab, resulting in disease progression after treatment.

Pyrotinib is an irreversible tyrosine kinase inhibitor targeting both HER2 and the epidermal growth factor receptor (EGFR), directly acts on the tyrosine kinase domain of the HER2 pathway, and completely blocks downstream pathways activated by homodimers or heterodimers of EGFR, HER2, and HER4 on tumor cell membranes (26). Pyrotinib in *HER2*-mutated non-small cell lung cancer (NSCLC) patients was found to display an objective response rate of 53.3% and a median PFS of 6.4 months (27). Promising antitumor activity and an acceptable safety profile in chemotherapy-treated patients with *HER2*-mutant NSCLC has also been reported (28). Pyrotinib in combination with capecitabine has been approved by the China National Medical Products Administration (NMPA) for patients with HER2-positive, advanced, or metastatic breast cancer that have previously received anthracycline or taxane chemotherapy. Pyrotinib treatment led to a median PFS of 8.00 months and a median OS of 19.07 months in HER2-positive metastatic breast cancer (29). Pyrotinib combined with capecitabine was determined to significantly improve objective response rate (ORR) (78.5 vs. 57.1%) and to prolong progression-free survival (PFS) (18.1 vs. 7.0 months) in patients with HER2-positive advanced breast cancer. Based on the results of pyrotinib for HER2-positive metastatic breast cancer in the clinical trials (30, 31), which aimed to explore the efficacy of pyrotinib in the treatment of HER2-overexpressed breast cancer. Because targeting HER2 has been a pivotal treatment strategy for HER2-positive breast cancer, the strategy has potential promise for treating HER2-positive ovarian cancer. Importantly, a case of successful treatment with pyrotinib for HER2-positive ovarian cancer (32). We have a surprising treatment result of using pyrotinib on an ovarian cancer patient whose HER2 study was positive. These encouraging results deserve further investigation to determine their impact on overall survival in patients with advanced or recurrent ovarian cancer who overexpress HER2.

Patients have also benefited from pyrotinib regardless of prior trastuzumab administration (33). Huang et al. (34) additionally revealed that for HER2-positive advanced gastric cancer patients who have developed a resistance to trastuzumab, pyrotinib is a promising new treatment that can be used as salvage therapy. In a recent case, a patient treated with pyrotinib yielded a PFS of 28 months, a promising result for the use of pyrotinib in treating HER2-positive ovarian clear cell carcinoma (32). Whether or not pyrotinib is effective in trastuzumab-resistant MOC is currently unclear. Bevacizumab is an angiogenesis inhibitor targeting VEGF, which results in the regression of tumor vasculature and the reduced formation of new blood vessels, leading to the suppression of tumor growth. Hamada-Nishimoto et al. have revealed that bevacizumab combined with paclitaxel is potentially effective in the treatment of HER2-positive metastatic breast cancer, if standard HER2-targeted therapy fails (35). Moreover, Gao et al. have indicated that EGFR or HER2 alterations are negative predictors of PFS for ovarian cancer patient treated with bevacizumab plus chemotherapy. Therefore, the clinicians may consider to use alternative regimens such as anti-EGFR or anti-HER2 targeted therapy instead of bevacizumab -based regimens on these patients when standard care fail (36). Despite the fast development of targeted therapies against other oncogenic drivers, treatments targeting *HER2* mutations in MOC are poorly described. Herein, we reported the case of a patient with *HER2* amplification recurrent MOC that had previously been treated with trastuzumab, and that benefited from pyrotinib and bevacizumab. Combined bevacizumab plus pyrotinib in ovarian cancer may play a synergistic role, but the specific mechanism of action in future studies we will study in depth.

In conclusion, we reported a case of recurrent MOC with *HER2* -amplification progression following a trastuzumab-based regimen, where a significant benefit using pyrotinib combined with capecitabine and bevacizumab, and a long-term clinical benefit was obtained, thus far, been determined for 22 months. Our case is an exploratory study for the use of pyrotinib in *HER2*-amplification MOC. The efficacy of pyrotinib in HER2-positive ovarian cancer should be further elucidated in large-scale clinical trials. Until such large-scale clinical trials are performed, our case report may provide valuable treatment information for patients with advanced or recurrent MOC.

## Data availability statement

The raw data supporting the conclusions of this article will be made available by the authors, without undue reservation.

## Ethics statement

This study has been approved by Ethic Committee of the Shulan (Hangzhou) Hospital Affiliated to Zhejiang Shuren University, Shulan International Medical College. The patients/participants provided their written informed consent to participate in this study. Written informed consent was obtained from the individual(s) for the publication of any potentially identifiable images or data included in this article.

## Author contributions

XF, HM, QL, XY, XH, LW, and YW all participated in the management of this case. XF, HM, NY, LG, and QL were in charge of manuscript drafting and data collection. XF, HM, and QL did the modification. All authors contributed to the article and approved the submitted version.
